# Phenotype disruption of umbilical cord derived MSC by cyclic mechanical stretch and hyperoxia mediated by p21

**DOI:** 10.1038/s41598-025-22330-6

**Published:** 2025-10-07

**Authors:** Maurizio J. Goetz, Judith Behnke, Frank Oehmke, Lena Holzfurtner, Pauline Korte, Stefano Rivetti, Saverio Bellusci, Harald Ehrhardt

**Affiliations:** 1 Department of General Pediatrics and Neonatology, Justus-Liebig- University Giessen and Universities of Giessen and Marburg Lung Center (UGMLC), Member of the German Center for Lung Research (DZL), 35392 Giessen, Germany; 2 Department of Gynecology and Obstetrics, Justus Liebig University of Giessen, Giessen, Germany; 3 Excellence Cluster Cardio Pulmonary Institute (CPI), Justus-Liebig-University Giessen and Universities of Giessen and Marburg Lung Center (UGMLC), Member of the German Center for Lung Research (DZL), 35392 Giessen, Germany; 4 Institute for Lung Health (ILH), 35392 Giessen, Germany; 5 Division of Neonatology and Pediatric Intensive Care Medicine, Department of Pediatrics and Adolescent Medicine, University Medical Center Ulm, Eythstr. 24, 89075 Ulm, Germany

**Keywords:** Lung disease, Mechanical ventilation, Hyperoxia, Mesenchymal stem cells, p21, Senescence, Mesenchymal stem cells, Respiratory tract diseases

## Abstract

Preclinical studies provided convincing evidence that umbilical cord derived mesenchymal stem cells (UC-MSC) prevent lung injury and promote lung regeneration. We hypothesized that cyclic mechanical stretch (CMS) and hyperoxia (HOX) during mechanical ventilation account for their limited therapeutic efficacy within the clinics. UC-MSC cultures were subjected to CMS and HOX and evaluated for proliferation, cell viability and further functional properties. Reversibility of the phenotype changes was evaluated after recovery in room air following these exposures. CMS and HOX compromised cell viability and proliferation, altered phenotypic characteristics, particularly PDGFRα expression, and induced cellular senescence in UC-MSC. Effects were most pronounced for CMS plus HOX. The alterations of UC-MSC were mediated by p21 accumulation. As inhibition of p21 aggravated cell death of UC-MSC, the results indicated a cell defense mechanism to ensure survival. This assumption was underpinned by the principal reversibility of the phenotype alterations and regrowth after removal of CMS and HOX. But prolonged strongest exposures resulted in definite phenotype changes. CMS and HOX have comparable effects on UC-MSC as described for lung resident MSC. Our results explain their timely limited presence in the diseased lung after therapeutic application. Future research should therefore focus on their repetitive application.

**Supplementary Information:**

The online version contains supplementary material available at 10.1038/s41598-025-22330-6.

## Introduction

For many acute and chronic diseases, only a limited number of efficient therapeutics are available that in most cases only alleviate severity of symptoms and disease progression. Mesenchymal stem cell (MSC) based therapies attracted particular attention during the last two decades due to their multiple benefits for the injured tissue and organs. They were judged particularly promising not only to alleviate tissue injury by inflammation and other damaging mechanisms but to preserve organ function and to induce tissue regeneration^1–4^. This research direction was derived from the observation that tissue stem cells are critical for physiologic tissue maintenance and repair following injury throughout the life span. By this, MSC therapy was prioritized as promising alternative or at least additional therapy for most of the common severe diseases across all ages^3–7^. The immunopriviledge of MSC compared to other types of stem cells derives from the fact that they do not induce a host immune response or cell rejection and the data from the clinical studies did not reveal any safety concerns^8,9^.

MSC therapy was extensively studied in the preclinical models of many acute and chronic lung diseases including acute lung injury, asthma, chronic obstructive pulmonary disease (COPD) and bronchopulmonary dysplasia (BPD)^9–11^. The beneficial effects of MSC therapy were mostly attributed to their anti-inflammatory and lung growth promoting properties while cell transdifferentiation and replacement of lung resident cells at the site of injury was of minor relevance^12–16^. MSC application was effective both in preventive models and during repair of lung injury. This is of importance when the deleterious insult occurs before a therapeutic intervention can be applied^9,17,18^. But disparities exist between the different lung diseases. For BPD, the benefits were still large after the end of hyperoxic injury. But for COPD, the therapeutic potential was restricted to the inflammatory phase of acute lung injury^18,19^. Therefore, MSC therapy seems particularly promising when applied during the acute phase of injury. Therapeutic efficacy was demonstrated for the various injuries including oxygen toxicity, mechanical ventilation and lung infectious diseases^9,17,20^.

Studies with MSC were executed with cells derived from the bone marrow, peripheral blood, umbilical cord blood, the Wharton’s jelly of the umbilical cord, the placenta and adipose tissue. They included cells from newborn and adult donors. In summary, MSC from the umbilical cord have the highest therapeutic potency^7,9,17,21,22^. And UC-MSC seem particularly suited to treat the diseased lung^3^. Both the topical application into the injured lung and the systemic application using venous or intraperitoneal injection proved efficacy^6,9,12–15^.

Unfortunately, the high efficacy in the preclinical models was not confirmed in the clinical trials on various lung diseases besides severe ARDS and in particular COVID-19 infection so far although for many other disease entities positive effects were seen^9,23–25^. Here, variations in the quality of product preparations now required in a multiple scaled volume, the dosage per kilogram of body weight, the use of fresh or frozen cell products, the differences in administration and the interaction with the lung environment might have impacted the results. But it must be stated that it is an ongoing realistic concern that the highly promising results from the preclinical models executed under ideal conditions in rodents cannot be reproduced in mankind with the multiple contributors to the disease within the real-life scenario and further disease contributors that cannot be respected in the preclinical models^4,26–28^. Observational studies in the different models using fluorescence-stained MSC indicated that MSC are present at the site of injury only for a short period of time which on the one hand is important not to have later adverse effects including the induction of malignancy but on the other hand limits the duration of therapeutic efficacy^9,29,30^. In that direction, some preclinical studies proved therapeutic superiority when MSC were applied repetitively^4,31^.

Hyperoxia (HOX) induces more pronounced lung cell injury with increasing oxygen concentrations of HOX that gets aggravated by the exposure to cyclic mechanical stretch (CMS) as correlate of mechanical ventilation^32–34^. Based on this knowledge, this study tested the hypothesis that the exposures of CMS and HOX exposure to the injured and inflamed lung account as well for the limited therapeutic efficacy of exogenously applied MSC within the clinics. We used a sophisticated in vitro model with exposure of UC-MSC to CMS and HOX and studied the effects on cell phenotype. Specifically, PDGFRα, αSMA and p21 were selected as marker proteins for MSC functionality in the injured lung^35^. Furthermore, we specified the molecular signaling pathway alterations responsible for the phenotype changes of UC-MSC provoked by the exposures.

## Materials and methods

### Cell culture and experimental settings

UC-MSC cultures were established as described by others before^18,36^. Umbilical cord samples from 18 healthy newborns delivered between 32 + 0 and 40 + 0 week’s gestation by cesarean section without any clinical signs of infection were used. Patient baseline characteristics are shown in Table 1. Umbilical cord segments of 10 cm in length were aseptically collected in theatre and stored in 25 ml of saline (PZN 2737779, B. Braun, Melsungen, DE) at 4 °C for up to 48 h. Under a sterile bench, vessels were rinsed with 4 °C PBS (#70011036, Thermo Fisher, Waltham, US). Samples were dissected into pieces of 3 cm in length and opened longitudinally. Umbilical cord vessels were removed. The remaining tissue was cut into quadratic pieces of 1–2 mm side length. 30 pieces were placed upside down on a 100 mm petri dish (#833902, Sarstedt, Nümbrecht, DE) and were left dry for 5 min to facilitate adhesion. Finally, tissue pieces were covered with 6.5 ml culture medium. MesenCult (#05401, Stemcell Technologies, Vancouver, CA) with 10% fetal calf serum (#10270106, Thermo Fisher, Waltham, US), 2% penicillin-streptomycin (#15140122, Thermo Fisher, Waltham, US) and 2% gentamycin (PZN 03928174, Ratiopharm, Ulm, DE) was used as culture medium in passage 0 (P0). First passaging was performed at 75% confluency after 16–18 days. Shortly before passaging, tissue pieces were removed through aspiration.

**Table 1:** Demographics of the study collective (*n* = 18).

Variables	Mean (IQR) or *n* (%)
GA (weeks)	38 + 2 (37 + 1–38 + 3)
BW (g)	3112 (2787–3430)
Male	9 (50.0)
Multiples	1 (5.6)
SGA	3 (16.7)
Indication	
Breech presentation	3 (16.7)
Placental abruption	2 (11.1)
Previous cesarean section	6 (33.3)
Macrosomia	1 (5.6)
Maternal request	5 (27.8)
Preeclampsia	1 (5.6)

Perinatal characteristics of the *n* = 18 newborn infants whose umbilical-cord derived MSC cultures were used in the study. Data are presented as mean (IQR; interquartile range) or n(%).

GA, gestational age; BW, birthweight; SGA, small for gestational age.

Cell culture conditions were conducted as described previously for lung resident MSC^37^. Culture medium was exchanged every 48–72 h. For experimental purposes, 200.000 cells from passage 1 (P1) were seeded onto each well of a collagen coated 6 well plate (#BF-3001 C, Flexcell, Burlington, US) with 3 ml culture medium each. From P1 onwards, MEM (#41090028, Thermo Fisher, Waltham, US) supplemented with 10% fetal calf serum (#10270106, Thermo Fisher, Waltham, US), 2% penicillin-streptomycin (#15140122, Thermo Fisher, Waltham, US) and 2% gentamycin (PZN 03928174, Ratiopharm, Ulm, DE) was used as culture medium.

Hyperoxia (HOX) was applied at 40% or 80% of oxygen relying on self-constructed chambers and constant oxygen concentrations with a maximum variation of ± 5% that were monitored using a GOX 100 oxygen sensor ((#GOX-100, Greisinger electronic GmbH, Regenstauf, Germany) and maximum deviations of 2.5% below and above were allowed^35^. Cyclic mechanical stretch (CMS) was executed with a cell stretching system (#Tissue Train, Flexcell, Burlington, US) and sinusoidal elongation of 1–8% at a frequency of 1 Hz and a duty cycle of 40% was used reflecting tidal volumes of about 10 ml per kg body weight.

siRNA transfection was performed as described before^37^ using Lipofectamine RNAiMAX (#13778150, Thermo Fisher, Waltham, US), p21 siRNA (#4390824-s417, Thermo Fisher, Waltham, US), and mock siRNA (#4390843, Thermo Fisher, Waltham, US). siRNA was applied 24 h prior to the start of the experiments at a concentration of 8.3 nM. For all experimental settings, experiments were repeated using independent biological replicates on different donor-derived UC-MSC to confirm reproducibility. Blinding of the evaluators to the applied treatment was not feasible as the exposures induced characteristic morphologic changes of the UC-MSC.

Cellular senescence staining was performed with β-galactosidase staining according to the manufacturer’s instructions (#9860S, Cell Signaling, Danvers, US).

All experiments with human MSC were performed in accordance with the principles of the Helsinki declaration and approved by the ethics committee of the Justus-Liebig-University Gießen (Az. 135/12). Written informed consent was obtained from the parents of all participating infants. Trial registration was done at Deutsches Register Klinische Studien (DRKS00004600).

### Cell expansion index

The cell expansion index (CEI) was calculated as the quotient of [cell count at the end of the experiment/ cell count at the start of the experiment] using manual cell counting of image recordings. Thereof, the change in CEI by the exposures of CMS and/or HOX was calculated compared to the spontaneous proliferation as (CEI exposure / CEI control) – 1. Thereby, the CEI reflects the increase in the number of cells from the start to the end of the observation.

### Flow cytometry

Flow cytometry was conducted on a BD Canto II flow cytometer (Becton Dickinson, Franklin Lakes, US) using FACS Diva (#FACS Diva 6.1.3, Becton Dickinson, Franklin Lakes, US) for data acquisition and FlowJo (#FlowJo 10.7.1, Becton Dickinson, Franklin Lakes, US) for data visualization. For cell quantification, count beads measuring 10 μm in diameter (#ACFP100-3, Spherotec, Lake Forest, US) were used. We performed cellular phenotyping using the following fluorochrome-labeled anti-human antibodies for surface staining, according to the manufacturer’s instructions: CD146 (361008, Biolegend, San Diego, US, conjugation: PE-Cy7), CD90 (328114, Biolegend, San Diego, US, conjugation: APC), CD73 (562430, Becton Dickinson, Franklin Lakes, US, conjugation: BV421), CD105 (323215, Biolegend, San Diego, US, conjugation: PerCP), CD45 (560777, Becton Dickinson, Franklin Lakes, US, conjugation: V500), CD165 (392040, Ancell, Bayport, US, conjugation: FITC), CD34 (343513, Biolegend, San Diego, US, conjugation: APC-Cy7) and CD11b (Becton Dickinson, Franklin Lakes, US, conjugation: BV605). Isotype-matched control antibodies were ordered from the same companies. The staining time for surface antibodies and isotypes was 30 min, followed by washing with staining buffer (1 × PBS/1% Albumin V; 70011036 Thermo Fisher, Waltham, US; 80764, Carl Roth, Karlsruhe, DE). Staining was performed on ice and MSC incubated with primary antibodies at the recommended concentrations. For viability measurement, Annexin V (#640947, Biolegend, San Diego, US) and Sytox (#S34857, Thermo Fisher, Waltham, US) stains were used. Prior to flow cytometry, cells were washed and resuspended in Annexin V Binding Buffer (#422201, Biolegend, San Diego, US). Incubation time was 30 min for count beads and Annexin V stain and 2 min for the Sytox dye. For quantification, absolute apoptosis was calculated as [dead cells/total frequency of single cells].

### Western blot

Cells were lysed using RIPA lysis buffer (#sc-24948, Santa Cruz Biotechnology, Dallas, US). Protein concentration was determined using a BCA assay (#23225, Thermo Fisher, Waltham, US) according to the manufacturer’s instructions and a Nanodrop (#ND-1000, Thermo Fisher, Waltham, US) device.

For SDS-PAGE, samples were diluted 1:1 with Laemmli loading buffer (#1610737, Biorad, Hercules, US). They were loaded to self-assembled 10% acrylamide gels. Running time was 75–90 min at a constant 40 mA current using a Powerpack (#Powerpack 300, Biorad, Hercules, US). For blotting, a semidry blot on a nitrocellulose membrane (#10600015, GE Healthcare, Chalfont St Giles, GB) was done using a Transblot Turbo system (#Transblot Turbo, Biorad, Hercules, US) at 25 V and 1 A for 30 min.

Blocking was done with dry milk 5% in TBS supplemented with 0.5% tween (TBS-T) at room temperature for 1 h. Incubation with the respective primary antibody was done overnight at 4 °C in the respective blocking buffer and with Albumin V 3% in TBS-T for PDGFRα antibody. Secondary antibody incubation was done for 1 h in dry milk 5% in TBS-T at room temperature. Primary antibodies were PDGFRα (#3174) and p21 (#2947S) both from Cell Signaling (Danvers, US), αSMA (#sc-58669, Santa Cruz, Dallas, US), and β-Actin (#ab-8227, Abcam, Cambridge, GB). HRP-conjugated secondary anti-rabbit (#7074S, Cell Signaling, Danvers, US) and anti-mouse (#sc-516102, Sant Cruz, Dallas, US) antibodies were used for signal detection.

Chemiluminescence was activated using an ECL reagent (#A38554, Thermo Fisher, Waltham, US) and detected in an imager (ChemiDoc XRS+, Biorad, Hercules, US). For sequential antibody stains, a stripping buffer was used (#21063, Thermo Fisher, Waltham, US).

Image processing was done in ImageLab (#ImageLab 6.0, Biorad, Hercules, US). Lane normalization factor was calculated as [observed signal of housekeeping protein for each lane/highest observed signal of housekeeping protein on the blot] and adjusted total band volume was normalized to β-Actin as [observed experimental signal/lane normalization factor]. Finally, sample volumes were again normalized to the volume of the respective control sample [normalized volume of each sample/normalized volume of control sample]. The order of samples in some blots was rearranged for the clearness of presentation without any further manipulation (indicated by separating lines). Full-length blots/gels are presented in Supplementary Fig. 1.

### Statistical analysis

For statistical purposes, data are given as median, interquartile range and minimum/maximum with box-and-whisker plots. Testing for statistical significance was done to compare the exposures and controls and exposure situations during mock and targeted siRNA transfection respectively that were done with two-sided, paired t-tests on a 5% significance level after testing for the normality of the data with the Shapiro-Wilk test. Statistical analysis was performed in R (#R 4.1.3, The R Foundation, Wien, AT) and Sigma Plot 12.3. (Systat Software, San Jose, CA).

## Results

We mimicked the intrapulmonary milieu of application of UC-MSC to patients with severely injured lungs that depend on mechanical ventilation and/or supplemental oxygen relying on our experimental in vitro setting with MSC and exposure to three-dimensional cyclic mechanical stretch (CMS) and/or hyperoxia (HOX) refined for studies on UC-MSC. UC-MSC from *n* = 18 healthy newborns delivered between 32 + 0 and 40 + 0 week’s gestation by cesarean section without any signs of infection were included into this work (Table 1).

### CMS and HOX lead to reduced viability and cell phenotype change in UC-MSC

**Fig. 1:**
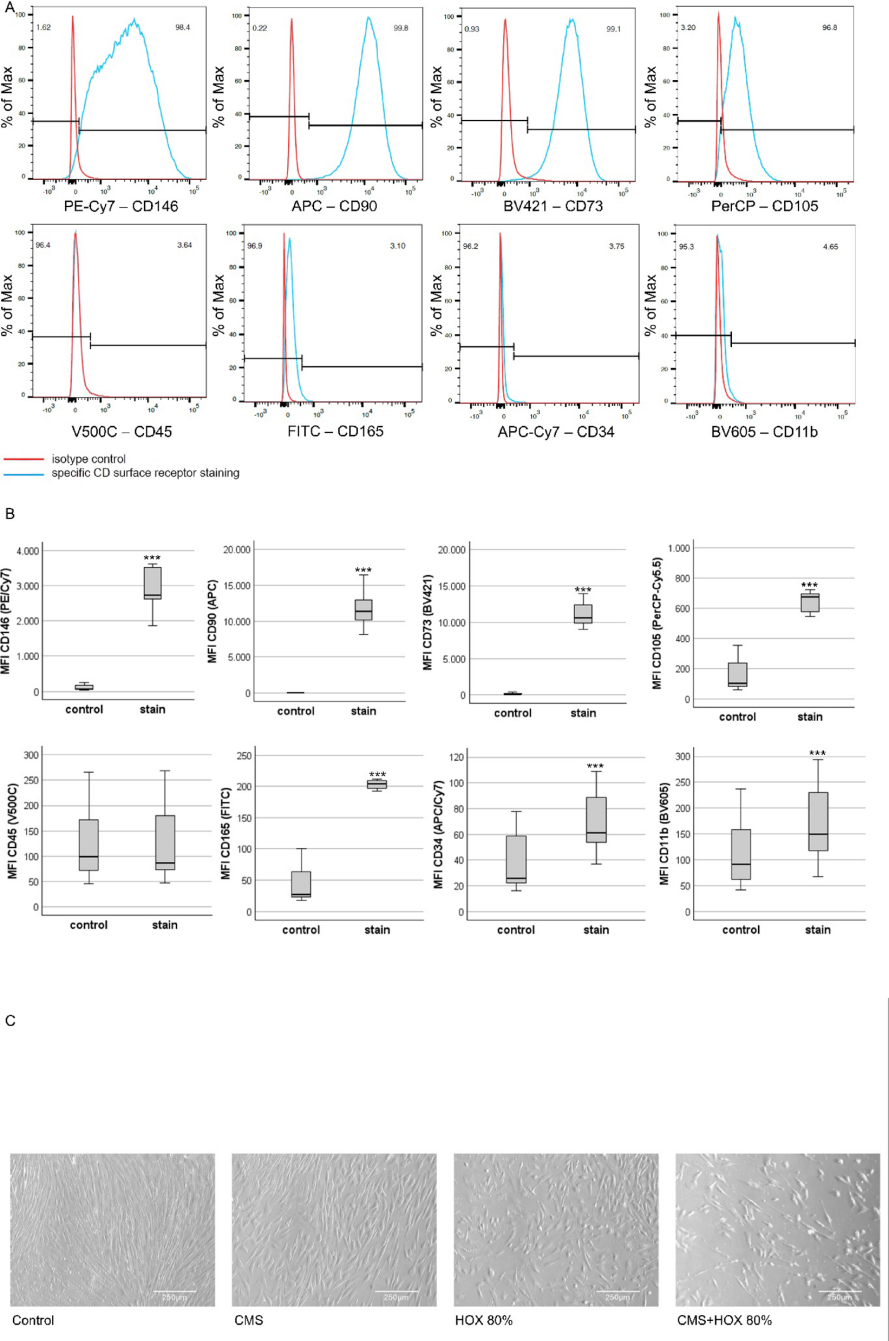

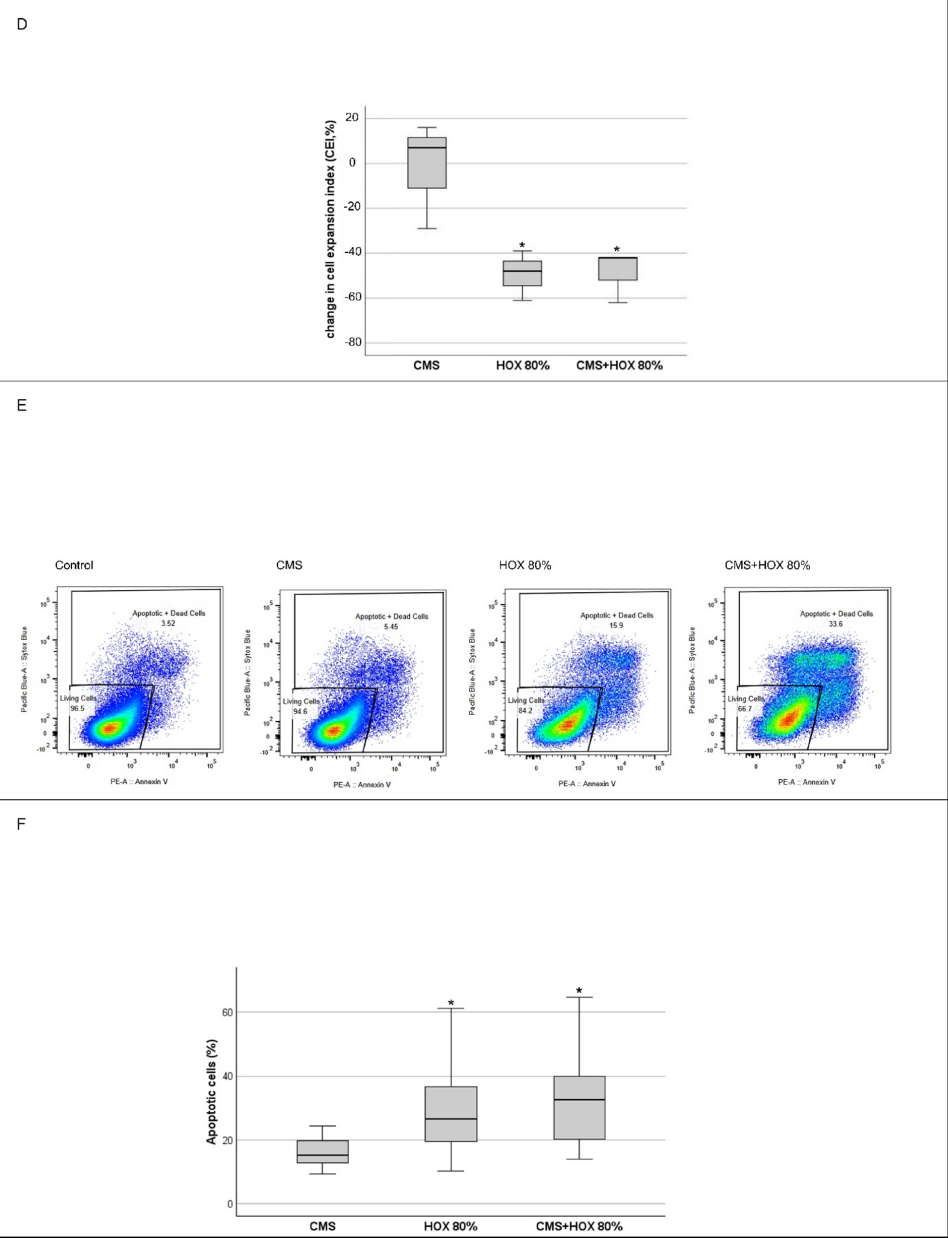
Single and combined treatment effect of cyclic mechanical stretch (CMS) and hyperoxia (HOX) on human umbilical cord derived MSC (UC-MSC) from newborn infants resulting in reduced proliferative capacity and cell viability. (**A**) Characterization of CD surface markers of MSC isolated from umbilical cords of newborn infants. Multicolor flow cytometry revealed expression of MSC-characteristic surface markers CD146, CD90, CD73 and CD105 whereas the hematopoietic marker CD45 was absent, and CD165, CD34 and CD11b only weakly positive. Positive fraction of stained cells versus isotype. Representative data of *n* = 7 independent UC-MSC cultures experiments. Red = unstained control, blue = specific staining. PE-Cy7 = Phycoerythrin-Cyanine 7; APC =; Allophycocyanin; BV421 = Brilliant Violet 421; PerCP = Peridinin-Chlorophyll-Protein; FITC = Fluorescein isothiocyanate; APC-Cy7 = APC-Cyanine 7; BV605 = Brilliant Violet 605. (**B**) Quantitative FACS data (MFI ratio log10 of all *n* = 7 independent UC-MSC cultures included into the FACS analyses). (**C**) Bright-field microscopy of proliferation after 72 h of selective and combined exposure to CMS and/or hyperoxia (HOX 80%). Scale bar 250 μm. Representative data of *n* = 3 independent UC-MSC cultures experiments. (**D**) Change in cell expansion index (CEI) was calculated as the quotient of [cell count at the end of the experiment/ cell count at the start of the experiment] using manual cell counting of image recordings. CMS and/or hyperoxic exposure (HOX 80%) led to a dose-dependent inhibition of MSC proliferation. *n* = 3 independent MSC cultures. (**E**) Representative flow cytometry analysis of *n* = 12 independent UC-MSC cultures experiments with exposure to CMS and/or HOX (HOX 80%). Live/dead staining using Annexin V/SYTOX Blue double staining and flow cytometry analysis. Presented is the absolute cell death (%) = dead cells / frequency of single cells. (**F**) Percentage increase of absolute apoptosis induced by selective and combined treatment with CMS and/or HOX (HOX 80%) compared to unexposed UC-MSC. *n* = 12 independent MSC cultures. Data are expressed as box plot with median and interquartile range, *p < 0.05, *** *p* < 0.001, by paired t-test, comparing exposures to the control situation.

UC-MSC displayed the CD surface marker characteristic for mesenchymal stem cells (Fig. 1A and B). Inter-patient variability in UC-MSC characteristics were observed in line with a previous publication^37,38^. The proliferative capacity and CD surface marker characteristics are displayed in Table S1 and S2. UC-MSC were exposed to CMS or HOX at 80% for 72 h. Reduced proliferation (Fig. 1D) and altered cell morphology were easily detectable on light microscopic images (Fig. 1C). Reduction of total cell numbers and cell viability after CMS and/or HOX were objectified by flow cytometry analyses using cell counts and cell death stainings. The negative impact on total cell counts and cell viability was more pronounced when HOX 80% was applied while CMS had no significant effect and most prominent when HOX 80% was combined with CMS (Fig. 1E and F).

**Fig. 2:**
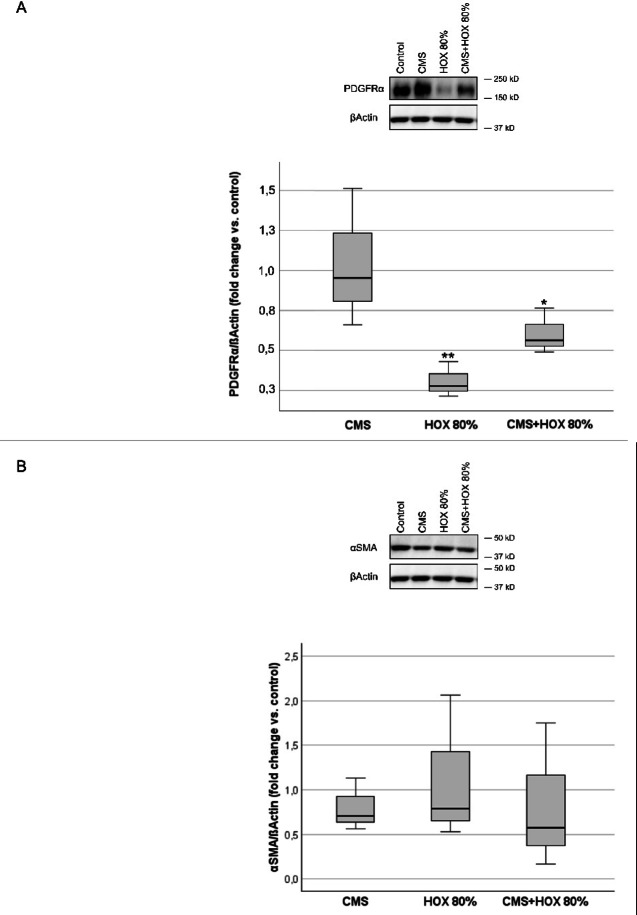

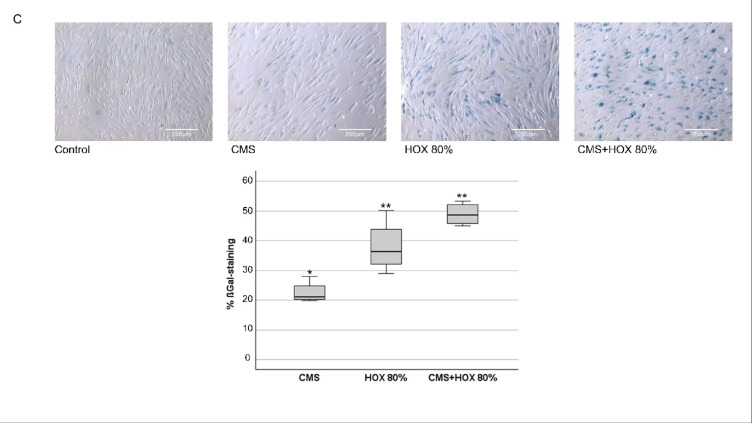
UC-MSC phenotype alterations by cyclic mechanical stretch (CMS) and hyperoxia (HOX). (**A + B**) Intracellular phenotype alterations in umbilical cord MSC (UCMSC) of newborn infants after exposure to CMS and/or HOX. (**A**) Western blot analysis showed reduced levels of PDGFRα after CMS and HOX 80% whereby HOX 80% and HOX 80% plus CMS demonstrated the largest effect size after 72 h treatment. *n* = 3 independent MSC cultures. (**B**) Western blot analysis showed no significant change in α-SMA expression after selective and combined treatment with CMS and HOX 80% for 72 h. *n* = 3 independent MSC cultures. The bar graphs show the densitometry results expressed as the ratio of adjusted total band volume normalized to ß-Actin and control. (**C**) CMS and/or HOX 80% increased cellular senescence–associated β-galactosidase (SA β-Gal) activity (blue; bright-field microscopy) after 72 h of exposure. Representative experiment of *n* = 4 independent MSC cultures. Scale bar 250 μm. Bar graph shows mean percentage change of β-Gal positive cells compared to unexposed UC-MSC. Data are expressed as box plot with median and interquartile range, *p < 0.05, **p < 0.01, by paired t-test, comparing exposures to the control situation. All full-length blots/gels from Figure 2 are presented in Supplementary Figure S1.

The exposure to CMS and/or HOX markedly reduced the PDGFRα protein level while α-SMA as marker of mesenchymal progenitor differentiation remained unchanged (Fig. 2A and B). As cell rounding and attenuated proliferative capacity represent typical features of cellular senescence and is typically induced following environmental damage and oxidative stress, we directed the further experiments to these studies^39^. Both CMS and HOX induced cellular senescence that was again most common for CMS plus HOX (Fig. 2C).

Taken together, we observed severe alterations in proliferation, viability and protein expression in UC-MSC after exposure to CMS and HOX. Therefore, we next studied the underlying signaling pathway alterations.

### p21 as mediator of UC-MSC fate following CMS and HOX exposure

**Fig. 3:**
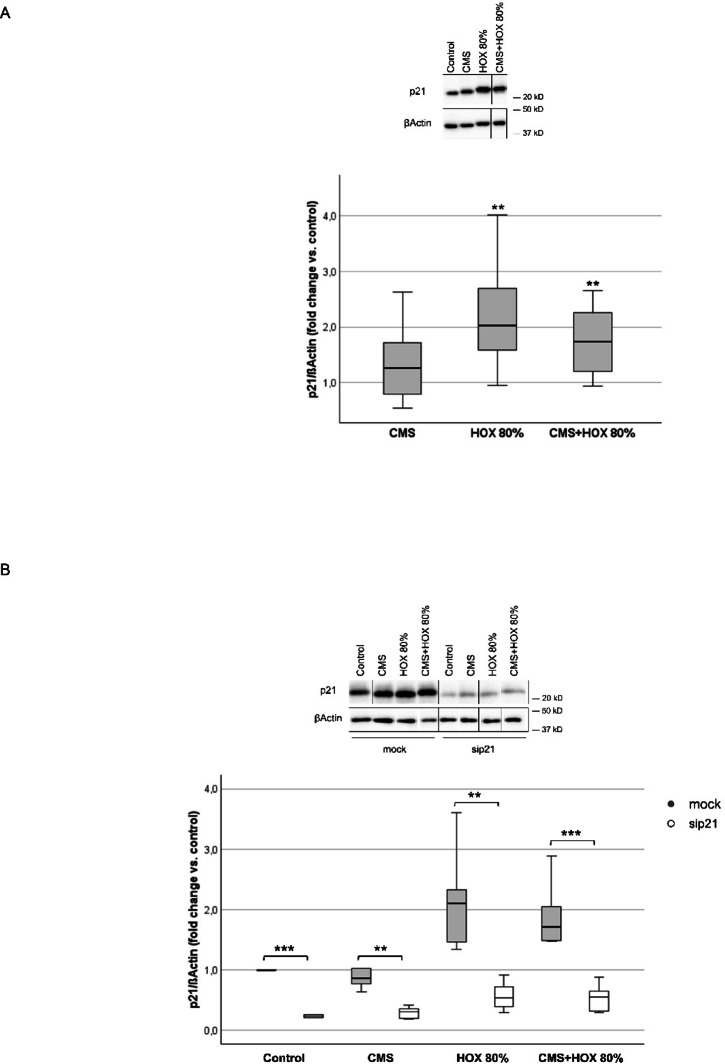

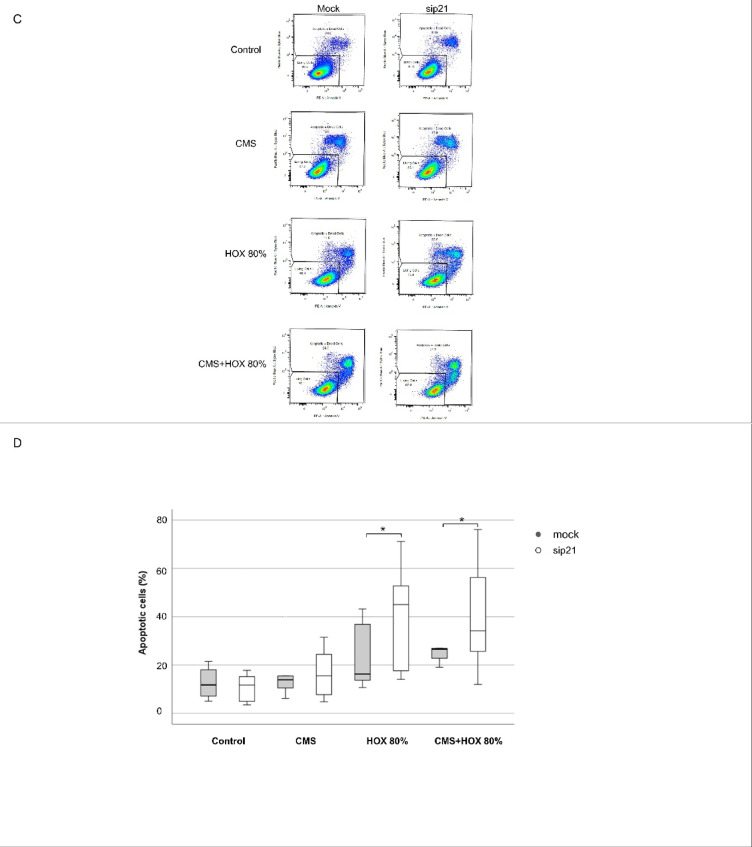
Activation of p21 in umbilical cord derived mesenchymal stem cells (UC-MSC) responsible for augmented apoptosis induction by cyclic mechanical stretch (CMS) and hyperoxia (HOX). (**A**) Representative western blot analysis of n=7 independent UC-MSC cultures experiments for p21 activation after 24h exposure to CMS and HOX 80%. CMS and HOX induced the accumulation of p21 whereas more pronounced for HOX and CMS+HOX, n = 7 MSC cultures. The order of samples was rearranged for the clearness of presentation without any further manipulation (indicated by separated boxes). The bar graphs show the densitometry results expressed as the ratio of adjusted total band volume normalized to ßActin and control. (**B**) Representative western blot analysis of n = 7 independent UC-MSC cultures of sip21 transfected cells show inhibited accumulation. The order of samples was rearranged for the clearness of presentation without any further manipulation (indicated by separated boxes). The bar graphs show the densitometry results expressed as the ratio of adjusted total band volume normalized to ßActin and unexposed UC-MSC for the n = 7 independent UC-MSC cultures. (**C**) Representative flow cytometry analysis of n = 7 independent UC-MSC cultures of each intervention group shows increase of apoptotic cell fraction in sip21 transfected UCMSC for each treatment with CMS and/or HOX 80%. Live/dead staining via Annexin V/SYTOX Blue flow cytometry analysis, absolute cell death (%) = dead cells / frequency of single cells. (**D**) Percentage of absolute apoptosis induction following selective and combined treatment with CMS and/or HOX (HOX 80%) of mock or p21 siRNA transfected UC-MSC by for the n = 7 independent UC-MSC cultures. Data are expressed as box plot with median and interquartile range, *p < 0.05, **p < 0.01, by paired t-test, comparing exposures to the control situation with specific siRNA against p21 to mock transfection respectively. All full-length blots/gels from this figure are presented in Supplementary Fig. S1.

To elucidate the signaling pathways leading to reduced UC-MSC viability and functionality, the previosuly described regulatory mechanisms of p21 accumulation in lung resident MSC were studied for the UC-MSC setting using western blot analyses. While p21 accumulation for CMS only did not demonstrated statistical significance, for HOX and the combined application a significant increase was detected (Fig. 3A).

To confirm the role of p21 as mediator of phenotype alteration and cellular senescence following CMS and/or HOX, inhibition studies were next executed applying RNA interference against p21. RNA interference against p21 reduced the number of viable UC-MSC when exposed to CMS and/or HOX and resulted in more pronounced apoptosis induction and the effect size was again strongest for CMS plus HOX 80% (Fig. 3B-D). These mechanistic investigations confirm p21 to be a cellular rescue mechanism to CMS and/or HOX application and fatal cell death induction.

### Reversibility of growth inhibition induced by CMS and/or HOX depending on the strength and duration of the exposures

**Fig. 4:**
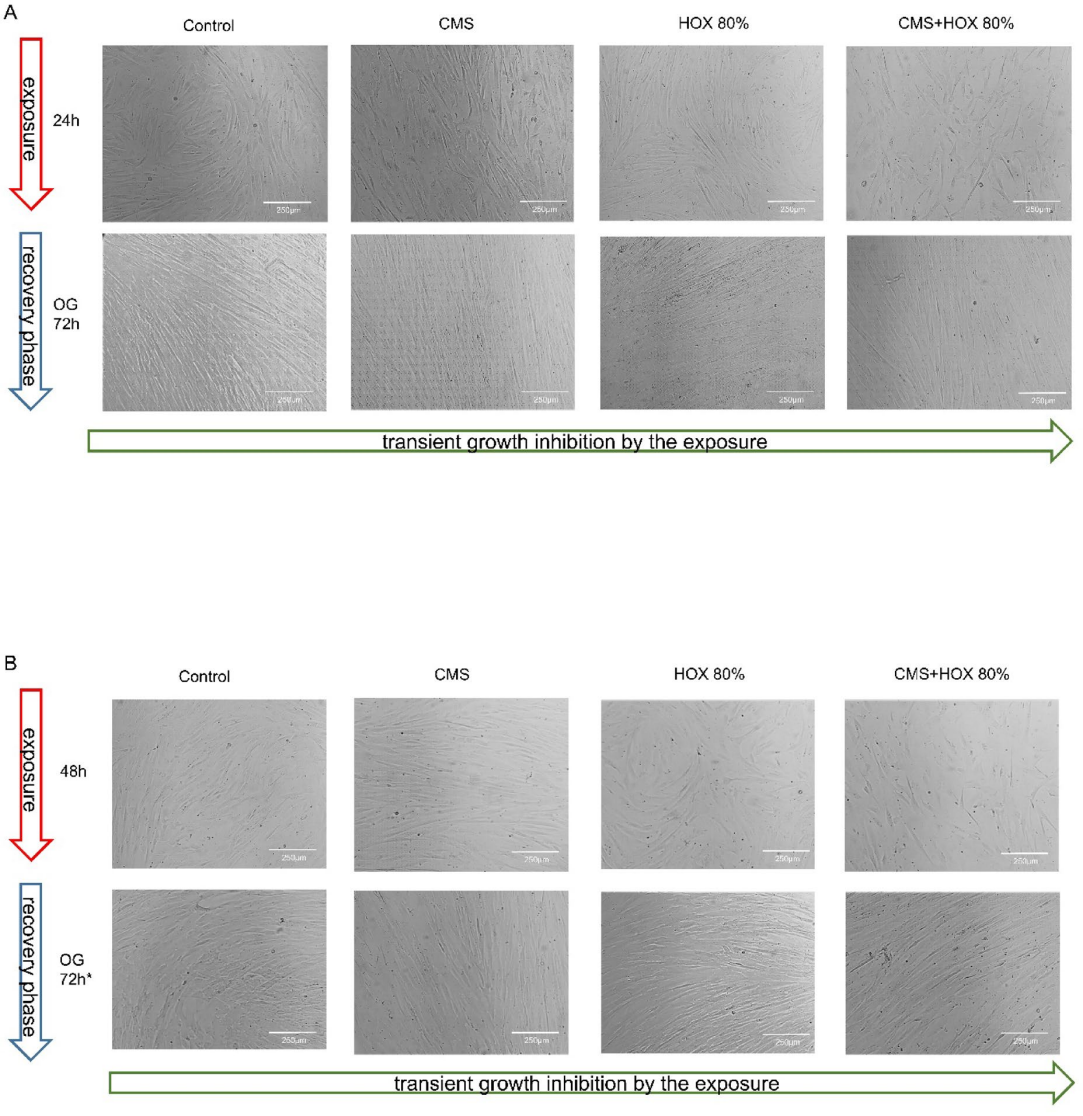

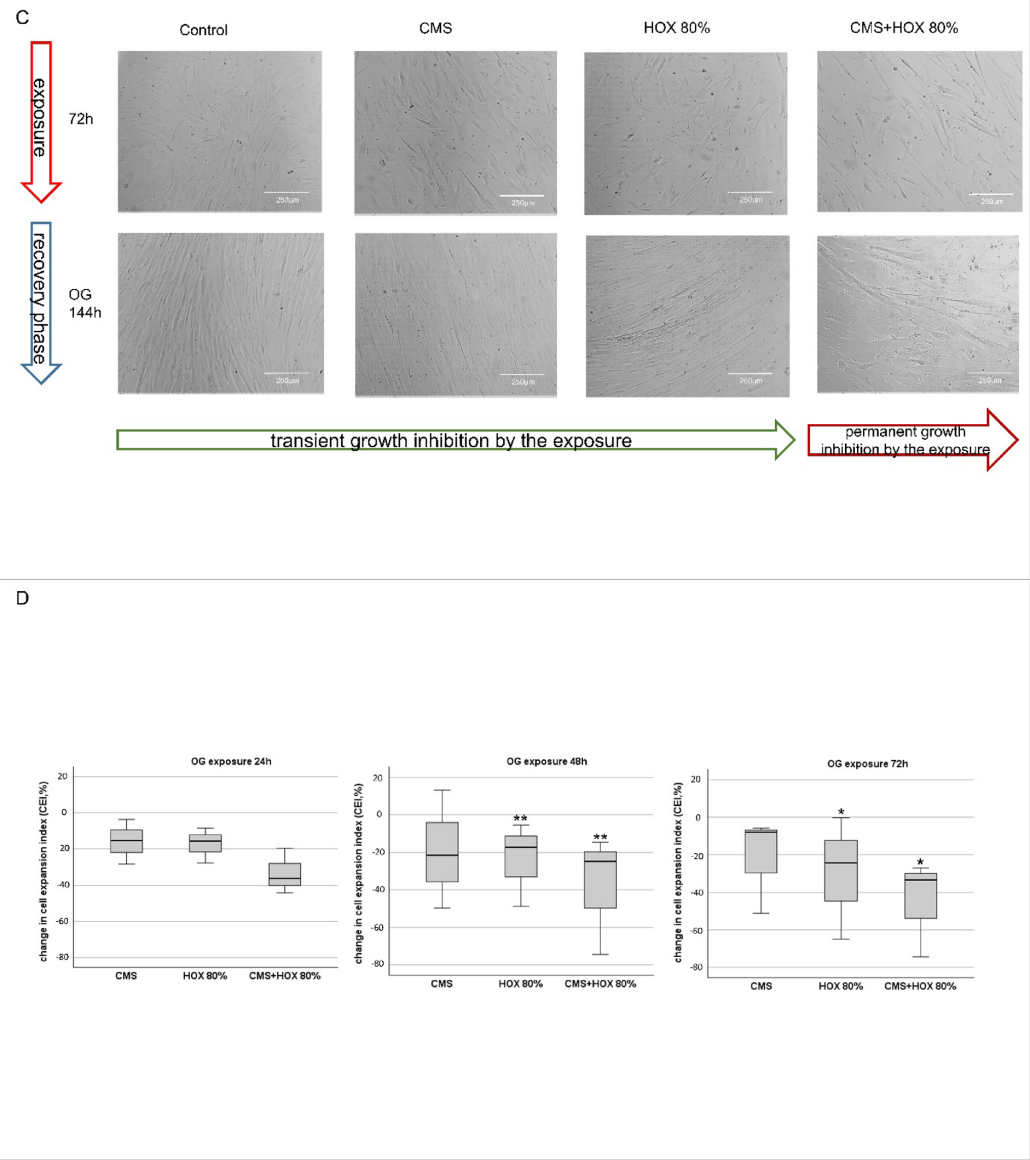
Reversibility of growth inhibition induced by cyclic mechanical stretch (CMS) and hyperoxia (HOX) in umbilical cord mesenchymal stem cells (UC-MSC) depending on strength and duration of the exposure (HOX 80%). (**A**) Bright-field microscopy of proliferation after 24 h of selective and/or combined exposure to CMS and HOX 80%. Outgrowth (OG) of cells in all intervention groups after further 3 days of recovery in room air without CMS. Scale bar 250 μm. (**B**) Bright-field microscopy of proliferation after 48 h of selective and/or combined exposure to CMS and HOX 80%. Outgrowth (OG) of cells in all intervention groups after further 3 days of recovery in room air without CMS (*after further 6 days in the HOX 80% and CMS + HOX 80% treatment group). Scale bar 250 μm. (**C**) Bright-field microscopy of proliferation after 72 h of selective and/or combined exposure to CMS and HOX 80%. OG of cells only in the selective treatment groups (CMS; HOX 80%) but not in combined treatment group (CMS + HOX 80%) after 6 days of recovery in room air without CMS. Representative experiments of *n* = 3. Scale bar 250 μm. (**D**) Change in cell expansion index (CEI) after 24 h/48 h/72 h of exposures was calculated as in Fig. 1D. CMS and/or hyperoxic exposure led to a time-dependent inhibition of MSC proliferation. Representative images of one experiment from *n* = 3 independent MSC cultures are displayed in A–C. In D, data are expressed as box plot with median and interquartile range, *p < 0.05, **p < 0.01, by paired t-test, comparing exposures to the control situation.

Lastly, we aimed to decipher the principal reversibility of the described growth arrest of UC-MSC exposed to CMS and/or HOX. After a 24, 48, or 72 h exposition, cells were further cultivated for another up to 6 days under baseline conditions. A 24 h application of CMS and/or HOX did not lead to a permanent growth inhibition (Fig. 4A). The 48 h exposures led to a transient growth inhibition for HOX and CMS + HOX intervention groups (Fig. 4B). Finally, a 72-hour application led to an irreversible growth inhibition for the CMS + HOX while growth inhibition for the CMS and HOX intervention groups was transient (Fig. 4C). Cell quantification via calculation of cell expansion index (CEI) is displayed in Fig. 4D.

These data allow the conclusion that the phenotype changes and UC-MSC growth arrest induced by CMS and HOX are in principle reversible but prolonged strong exposures result in permanent loss of renewed outgrowth capacity of UC-MSC. Therefore, the duration and strength of the exposures determines the further fate of UC-MSC.

## Discussion

In this study we demonstrate that cyclic mechanical stretch (CMS) and hyperoxia (HOX) induce identical phenotype alterations in UC-MSC after CMS and HOX as had been described for lung resident MSC before. The results are in congruency with a broad array of research on this topic where similar regulatory patterns and cell fates were described after exposure to physical stress or oxygen toxicity. The induction of cellular senescence via p21 has been established as a general mechanism following HOX injury and during MSC aging^40–43^. Notably, we are the first to describe the potential reasons for the restricted therapeutic potential of UC-MSC in clinical trials relying on a sophisticated in vitro experimental approach. Lastly, the results of our studies explain why the therapeutic efficacy is limited and application of exogenous UC-MSC to the injured lung results in the rapid disappearance of these cells. The downregulation of PDGFRα constitutes one of the most important phenotype alterations. This finding fits well into other published results on the functionality of MSC where suppression or loss of PDGFRα went along with loss of functionality and the production of lung growth promoting factors like VEGFA^44,45^.

Dysregulation of lung resident MSC has been ascribed a hallmark of lung pathology across a broad array of pulmonary diseases. The origins of lung disease pathologies are disparate but they all have in common a pronounced inflammatory response that gets aggravated by the therapeutic application of supplemental oxygen and mechanical stretch. This makes the lung particularly vulnerable to further stressors as the anti-inflammatory properties get out of balance and lung regeneration is no longer feasible^11,34,46^. The results from preclinical studies on UC-MSC therapies revealed tremendous benefits for the lungs and therefore researchers might have overseen the potential harms of the local lung environment following the intrapulmonary application of UC-MSC. Furthermore, the permanent phenotype change in UC-MSC was less pronounced in our model than in the previous studies on lung resident MSC and lower effect size can be another reason why this has not been detected before^35^. But the results on the rapid disappearance of UC-MSC at the site of injury underpin our hypothesis.

The presented results clearly indicate that there is a dose-response relation between the extent of UC-MSC dysfunctionality and the extent and duration of exposures. Not surprisingly, HOX toxicity was most devastating when combined with CMS. It must be stated that MSC derived from the umbilical cord stem from a hypoxic milieu in utero where their functional properties were probably best preserved besides the origin from younger donors compared to other sources of MSC. In line, hypoxic preconditioning was repeatedly demonstrated to boost the effectivity of MSC from other origin^47,48^. Here it needs to be acknowledged that even the application of UC-MSC to patients breathing room air constitutes a relatively hyperoxic environment compared to the source conditions. Furthermore, the inflammatory milieu in the diseased lungs is driven by further stimuli besides reactive oxygen toxicity including infectious stimuli that result in comparable activation of pathways of inflammation^9,26,27^. Future research approaches need to keep these aspects in mind.

Despite the convincing research results that fit into the vast scientific publications on the topic of injury induced by physical stress and oxygen toxicity, it must be stated that our results were solely obtained under in vitro cell culture conditions that do not reflect the complexity of the injured lung and we were not able to study the effects of RNA interference on cellular senescence to establish a direct link to p21. The results need to be reproduced in a larger sample size and demand the further confirmation and exploitation in more complex scenarios including infectious stimuli and co-culture of different lung cells e.g. within organoid models. And it will be important to address the inflammatory reactivity of the UC-MSC when exposed to the inflammatory stimuli of CMS and HOX. Lastly, in vivo cell lineage tracing will deliver better estimates of UC-MSC cell fate that was beyond the scope of our studies and it will be important to determine a true UC-MSC recovery versus the expansion of non-senescent UC-MSC subpopulations and to execute longitudinal time-course analyses. But the congruent results between the different experimental exposures and readouts and the matching of our finding to previous observations in lung resident MSC and other disease entities and different cell populations argue towards a general phenomenon^40,49–52^.

## Conclusions

Our data add novel mechanistic insights deciphering the effects of cyclic mechanical stretch (CMS) and hyperoxia (HOX) on UC-MSC phenotype and functionality. While disruption of the intracellular signaling cascade resulting in the observed phenotype alterations does not constitute a therapeutic option as it aggravates the injury resulting in cell death. We cannot rule out that alternative protective responses got activated including DNA damage response signaling or autophagy. Our data suggest a plausible contributing mechanism to clinical observations, but the link to attenuated clinical efficacy remains speculative pending further in vivo validation. Future studies should expand for further potency measures not assessed in this study to fully evaluate UC-MSC therapeutic potential in the clinical context, including ability for colony-formation and trilineage differentiation, immunomodulatory capacity and response to inflammatory priming. Our findings imply that the reduction of the extent and duration of exposures and the repetitive application of UC-MSC constitute suitable approaches to circumvent efficacy limitations and to cover the complete period of disease progression as is nowadays standard for many other therapeutic approaches. The implications of our results can reach beyond the lung and should encourage research activities intended to optimize MSC-based therapies to take our findings into consideration.

## Supplementary Information

Below is the link to the electronic supplementary material.

Supplementary Material 1

## Data Availability

The datasets used and/or analyzed during the current study are available from the corresponding author on reasonable request. All data generated or analyzed during this study are included in this published article and its supplementary information files. The supplementary material is available at the online public repository *figshare* via: 10.6084/m9.figshare.27241677.v5.
