# Supplementary material for: Phenotype disruption of umbilical cord derived MSC by cyclic mechanical stretch and hyperoxia mediated by p21
Source: Sci Rep. 2025 Oct 7;15:35031. doi: 10.1038/s41598-025-22330-6 (PMC12504700; doi:10.1038/s41598-025-22330-6)
Supplement: Supplementary file 1 — Supplementary Material 1 [file 41598_2025_22330_MOESM1_ESM.pdf]

SUPPLEMENTAL MATERIAL

Phenotype disruption of umbilical cord derived MSC by cyclic mechanical stretch and hyperoxia mediated by p21

Maurizio J Goetz[1*], Judith Behnke[1*], Frank Oehmke[2], Lena Holzfurtner[1], Pauline Korte[1], Stefano Rivetti[3,4], Saverio Bellusci[3,4], Harald Ehrhardt[1,5**]

[1] Department of General Pediatrics and Neonatology, Justus-Liebig-University Giessen and Universities of Giessen and Marburg Lung Center (UGMLC), Member of the German Center for Lung Research (DZL), 35392 Giessen, Germany.

[2] Department of Gynecology and Obstetrics, Justus Liebig University of Giessen, Giessen, Germany

[3] Department of Internal Medicine, Justus-Liebig-University Giessen and Universities of Giessen and Marburg Lung Center (UGMLC), Excellence Cluster Cardio-Pulmonary Institute (CPI), Member of the German Center for Lung Research (DZL), 35392 Giessen, Germany.

[4] Institute for Lung Health (ILH), 35392 Giessen, Germany.

[5] Division of Neonatology and Pediatric Intensive Care Medicine, Department of Pediatrics and Adolescent Medicine, University Medical Center Ulm, 89075 Ulm, Germany.

* both authors contributed equally

Supplemental Table S1: Quantitative FACS data (MFI and MFI ratio of all n=7 preterm infants included into FACS analyses)

ID	*CD11b (BV605)*		*CD34 (APC/ Cy7)*		*CD45 (V500C)*		*CD73 (BV421)*		*CD90 (APC)*		*CD105 (PerCP-Cy5.5)*		*CD146 (PE/Cy7)*		*CD165 (FITC)*	
	**control**	**stained**	**control**	**stained**	**control**	**stained**	**control**	**stained**	**control**	**stained**	**control**	**stained**	**control**	**stained**	**control**	**stained**
1	2,375	2,468	1,892	2,037	2,425	2,430	2,642	4,012	2,155	3,909	2,548	2,861	2,403	3,430	2,000	2,423
2	2,246	2,382	1,833	2,013	2,281	2,314	2,473	3,979	2,149	4,054	2,439	2,831	2,290	3,270	1,863	2,305
3	2,149	2,342	1,699	1,869	2,185	2,188	2,358	4,145	2,090	3,992	2,301	2,785	2,149	3,557	1,732	2,310
4	1,959	2,173	1,415	1,785	1,996	1,934	2,104	4,033	1,833	4,017	2,013	2,848	1,869	3,535	1,431	2,314
5	1,699	2,017	1,322	1,690	1,813	1,820	1,959	4,145	1,771	4,216	1,881	2,737	1,740	3,437	1,362	2,283
6	1,623	1,833	1,204	1,568	1,663	1,672	1,820	4,024	1,653	4,136	1,763	2,597	1,623	3,408	1,230	2,241
7	1,869	2,121	1,362	1,771	1,898	1,908	2,013	3,954	1,851	4,088	1,924	2,836	1,778	3,697	1,362	2,326

Supplemental Table S2: Perinatal characteristics and proliferation index of n=6 newborn infants whose umbilical-cord derived MSC cultures were used in the study.

ID	*GA (weeks)*	*BW (g)*	*sex*	*multiple birth*	*SGA*	*PI*
1	38+5	3320	male	no	no	1,3
2	38+2	3010	male	no	no	2,8
3	40+0	3400	female	no	no	2,5
4	32+0	1450	female	no	no	2,8
5	38+5	3695	male	no	no	3,2
6	36+0	2170	male	no	yes	2,7

Proliferation index calculated was calculated as the quotient of [cell count at the end of the experiment/cell count at the start of the experiment] using manual cell counting of image recordings.
GA – gestational age; BW – birthweight; SGA – small for gestational age; PI – proliferation index

Supplemental Figure S1: Original Western Blot

Figure 2A

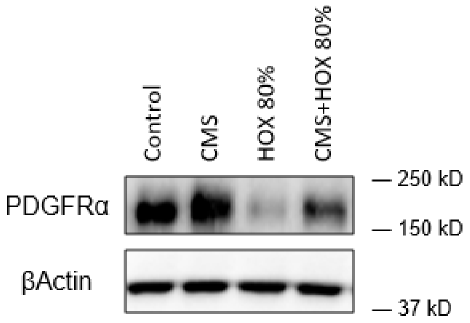

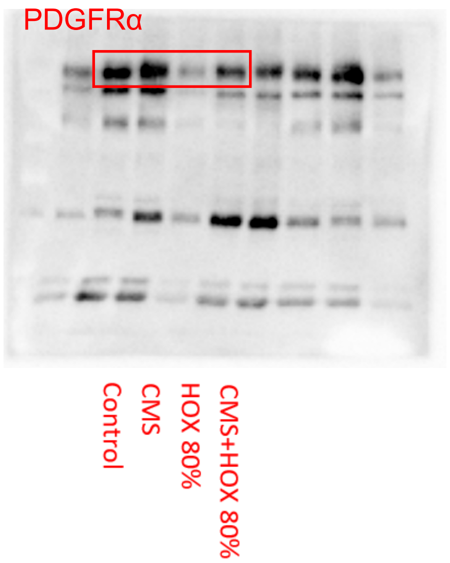

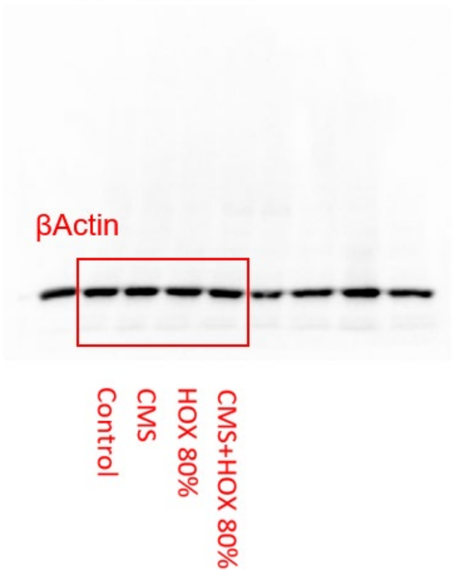

Figure 2B

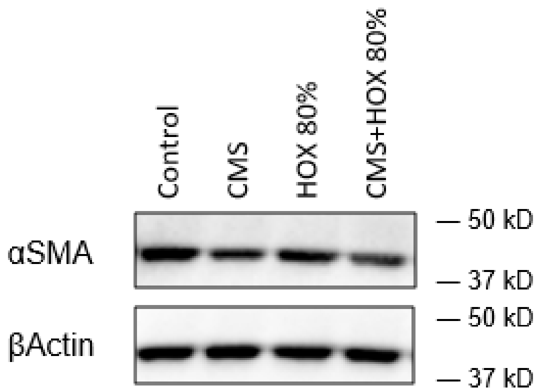

Control
CMS
HOX 80%
CMS+HOX 80%
αSMA
βActin
— 50 kD
— 37 kD
— 50 kD
— 37 kD

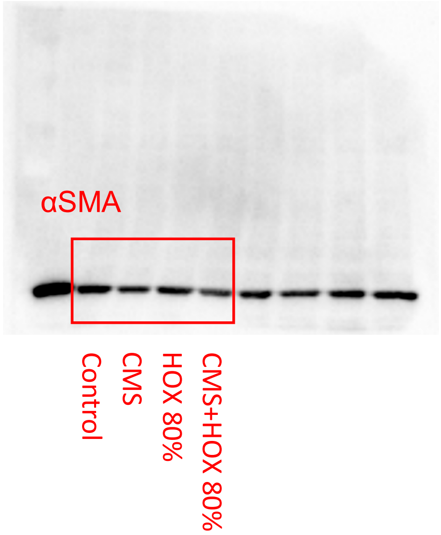

αSMA
Control
CMS
HOX 80%
CMS+HOX 80%

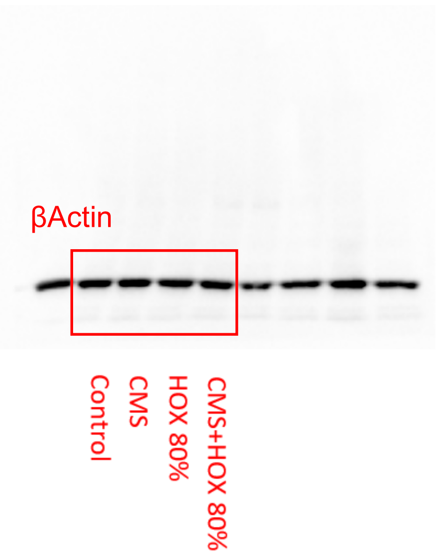

βActin
Control
CMS
HOX 80%
CMS+HOX 80%

Figure 3A

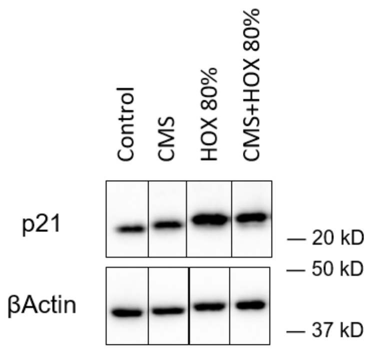
Control
CMS
HOX 80%
CMS+HOX 80%
p21
βActin
— 20 kD
— 50 kD
— 37 kD

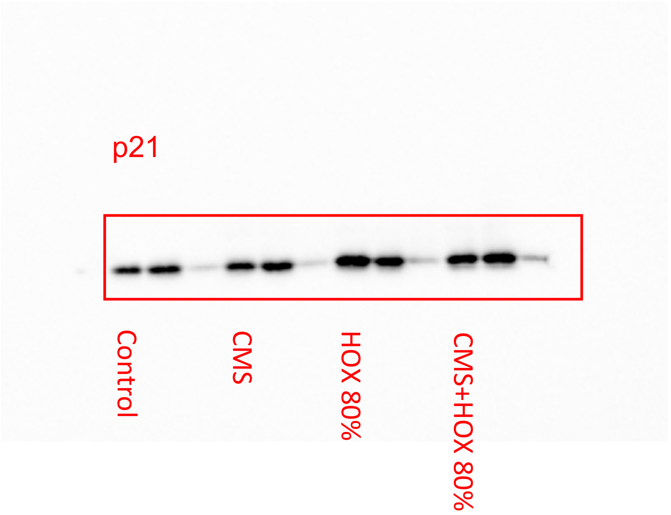
p21
Control
CMS
HOX 80%
CMS+HOX 80%

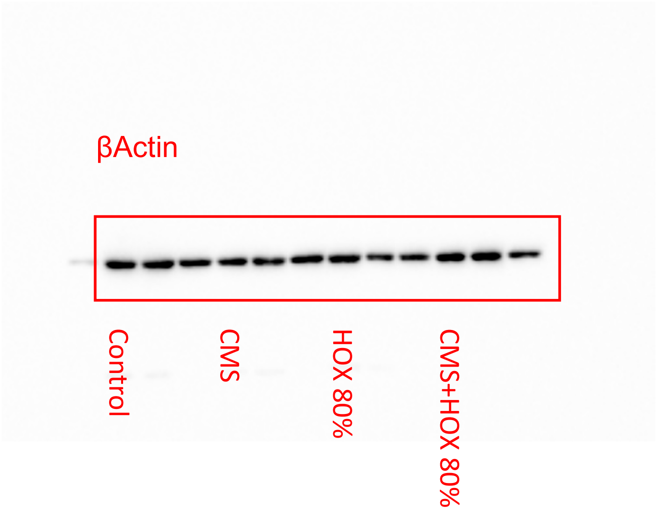
βActin
Control
CMS
HOX 80%
CMS+HOX 80%

Figure 3B

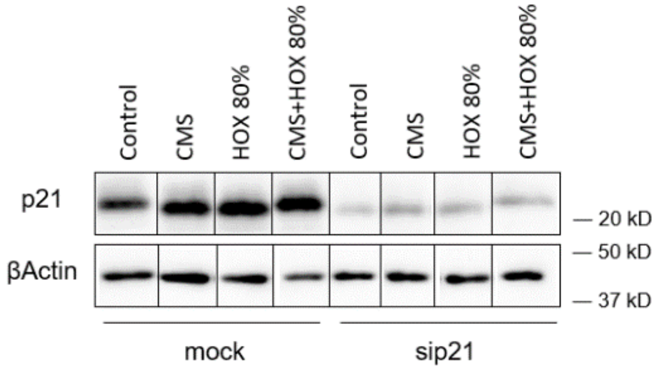
Control
CMS
HOX 80%
CMS+HOX 80%
Control
CMS
HOX 80%
CMS+HOX 80%
p21
βActin
— 20 kD
— 50 kD
— 37 kD
mock
sip21

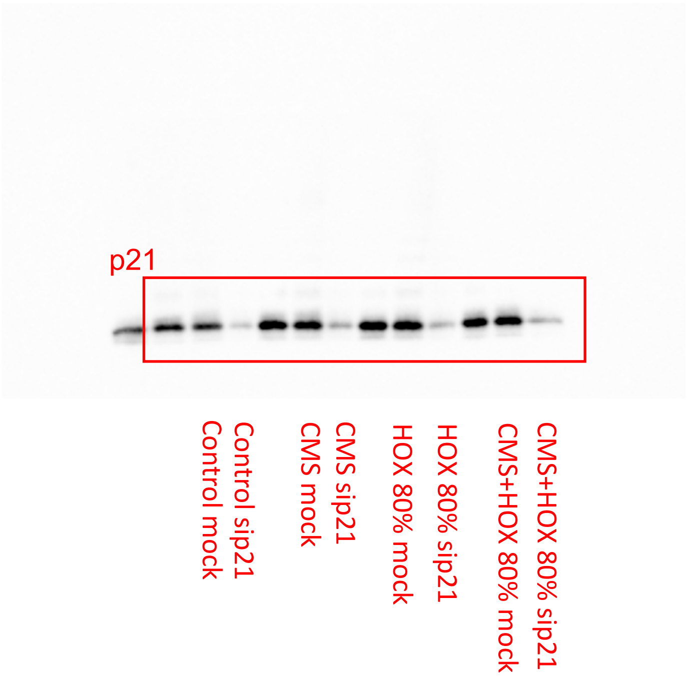
p21
Control mock
Control sip21
CMS mock
CMS sip21
HOX 80% mock
HOX 80% sip21
CMS+HOX 80% mock
CMS+HOX 80% sip21

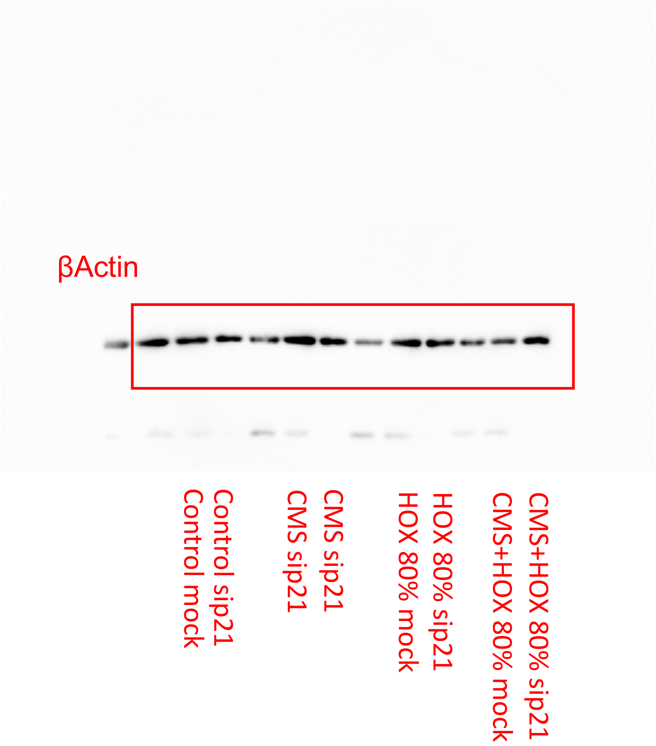
βActin
Control mock
Control sip21
CMS sip21
CMS sip21
HOX 80% mock
HOX 80% sip21
CMS+HOX 80% mock
CMS+HOX 80% sip21
